# Feasibility of atrial septal defect type II occluders determined by computed tomography virtual cardioscope

**DOI:** 10.3389/fradi.2026.1670748

**Published:** 2026-02-19

**Authors:** Mohamad Yanuar Amal, I-Lun Shih, Chiueng-Fang Wu, Jou-Kou Wang, Shyh-Jye Chen

**Affiliations:** 1 Division of Pediatric Radiology, Department of Radiology, Dr. Cipto Mangunkusumo General Hospital, Faculty of Medicine Universitas Indonesia, Jakarta, Indonesia; 2 Department of Medical Imaging, National Taiwan University Hospital, Taipei, Taiwan; 3 Department of Pediatrics, National Taiwan University Hospital, Taipei, Taiwan

**Keywords:** ASD occlusion therapy, CT virtual cardioscopy, morphological assessment, percutaneous transcatheter closure, type II ASD

## Abstract

**Objectives:**

This study aims to assess the suitability of large ASD type II occluders by evaluating the locations of the ASD and its extensions, as well as the adequacy of the rim, through the utilization of CT virtual cardioscopy.

**Background:**

Atrial septal defects (ASDs) represent the most frequent congenital heart abnormalities observed in adults and the second most prevalent in infants. There has been a notable paradigm shift over the past four decades which percutaneous transcatheter closure as the preferred treatment modality over traditional surgical method.

**Methods:**

We conducted a retrospective descriptive study on patients with type II ASD who undergone transcatheter and surgical closure from January 2007 to April 2023. Rigorous exclusion criteria were applied to ensure a homogeneous study population and enhance the validity of research findings. All MDCT measurements were performed independently in a blinded manner. Statistical analysis was performed using independent T-test and Chi-square.

**Results:**

A total of 24 patients were evaluated by virtual cardioscope. Eighteen patients underwent successful occlusion therapy for ASD without complications. Six patients underwent surgery to close the interatrial defect. Patients who undergone transcatheter approach were older than patients in the surgical group. There were no differences between the 2 groups in regard to long axis and short axis in MDCT (all *p* > 0.05). The rimless angle in the operation group is larger than the operation group and there were differences between the 2 groups (*p* < 0,05). Location and extension of the defect did not have significant association in determining treatment procedure.

**Conclusions:**

Accurate morphological assessment play a crucial role in assessing the suitability of either a percutaneous approach or surgical correction for atrial septal defects (ASD).

## Introduction

Atrial septal defects (ASDs) represent the most frequent congenital heart abnormalities observed in adults and the second most prevalent in infants, following ventricular septal defects. Left untreated, a large atrial septal defect (ASD) characterized by left-to-right shunting may induce right ventricular volume overload, leading to pressure overload. Consequently, this cascade of events can culminate in right ventricular failure, irreversible pulmonary hypertension, arrhythmias, and paradoxical embolization (1). The gold standard for treating adult patients with atrial septal defect (ASD) has historically been surgical closure. However, over the past four decades, there has been a notable paradigm shift in managing secundum-type ASDs. This shift has increased the adoption of percutaneous transcatheter closure as the preferred treatment modality over traditional surgical closure methods. Advancements in medical technology and the development of innovative devices and techniques have facilitated the evolution toward percutaneous transcatheter closure. These advancements have significantly improved the effectiveness and safety of transcatheter procedures, making them a viable alternative to surgery for many patients with secundum-type ASDs.

Currently, transcatheter closure is considered the treatment of choice for ostium secundum ASD, particularly in cases where the anatomical characteristics meet specific criteria. These criteria typically include a stretched diameter of less than 38 mm and a sufficient rim of at least 5 mm, except in areas adjacent to the aorta. The procedure must be hemodynamically feasible for optimal outcomes. As transcatheter closure demonstrates favorable results and benefits for patients with secundum-type ASDs, it is increasingly becoming the standard of care in interventional cardiology. The ongoing refinement of techniques and devices in this area underscores the importance of staying abreast of advancements to provide the best possible care for patients with ASDs (1).

During a four-year follow-up period, observational studies have revealed that percutaneous closure outperformed surgical repair in terms of early complications, atrial flutter/fibrillation, systemic thromboembolism, ischemic stroke, and overall mortality (1).

Although percutaneous device closure for atrial septal defects (ASD) is considered safe and effective, it is essential to acknowledge that there remains a potential for the occurrence of various complications. Operators performing these procedures may encounter obstacles when dealing with complex lesions that present unique challenges, such as multiple defects, rim deficiencies, or large defects. In such cases, healthcare providers require careful consideration and expertise to navigate these complexities and ensure successful outcomes for patients undergoing percutaneous closure (2).

Accurate measurements and detailed morphological delineation play a crucial role in assessing the suitability of either a percutaneous approach or surgical correction for atrial septal defects (ASD). Factors such as the precise location, quantity, and size of the defect and the lengths of the surrounding rims are essential considerations that inform the treatment decision-making process. By carefully evaluating these parameters, healthcare providers can tailor the treatment approach to best meet each patient's individual needs, ultimately optimizing the outcomes of ASD closure procedures (3). Studies have reported instances where device closure procedures have been guided exclusively by either echocardiography or fluoroscopy. Historically, transesophageal echocardiography (TEE) has been the predominant modality for providing echocardiographic guidance during atrial septal defect (ASD) closure interventions. This longstanding reliance on TEE underscores the significance of established protocols and the evolving landscape of imaging modalities in guiding interventional cardiology procedures within the healthcare domain (4).

However, it is essential to note that transesophageal echocardiography (TEE) may not comprehensively evaluate atrial septal defects (ASDs), especially in cases involving large defects or oval morphologies, compromising its accuracy. Moreover, it is essential to highlight that TEE is an invasive procedure with potential complications affecting the respiratory and gastrointestinal tracts. The stretched balloon diameter (SBD), considered the gold standard for determining device dimensions, also tends to magnify the defect (3).

However, determining the optimal device size should be personalized, considering factors such as heart size, inadequate margins, and the spatial relationship with neighboring cardiac structures. Large defects frequently coincide with deficiencies in the surrounding rims, increasing the likelihood of complications such as embolization, erosion, and device encroachment onto adjacent cardiac structures (3). Ensuring careful consideration of prosthesis rims and dimensions before embarking on percutaneous transcatheter closure of an ostium secundum atrial septal defect (ASD) is crucial in cardiac MDCT. With its capacity for volumetric imaging and exceptional spatial resolution, cardiac MDCT can offer a comprehensive anatomical assessment of ASD suitability for percutaneous closure. Moreover, cardiac MDCT plays a significant role in identifying any anomalies that could impede or contraindicate ASD repair, such as undiscovered partial anomalous pulmonary venous connections. Not only useful for pre-treatment, but cardiac CT imaging can also aid in evaluating the relationship between the occluder and intracardiac structures in ASD patients post-transcatheter closure. The advancements in image quality, encompassing high resolution and new software for three-dimensional post-processing, have facilitated non-invasive examinations of internal cavities (5). Computed tomography (CT) with virtual cardioscopy emerges as a non-invasive technique utilized for pre-procedural evaluation of patients. This study aims to assess the suitability of large ASD type II occluders by evaluating the locations of the ASD and its extensions, as well as the adequacy of the rim, through the utilization of CT virtual cardioscopy.

## Materials & methods

### Study population

The study implemented rigorous exclusion criteria to ensure a homogenous study population and improve the validity of the findings. Patients were excluded if they met any of the following conditions:

1. Incomplete or non-protocol multidetector computer tomography (MDCT) examination.
2. Previous surgical closure of the ASD.
3. Use of prosthetic devices other than secundum-type ASD occluders.
4. Absence of definitive treatment (transcatheter or surgical)
5. Incomplete imaging datasets that precluded measurement of rimless angles or quadrant classification.

**Figure 1:**
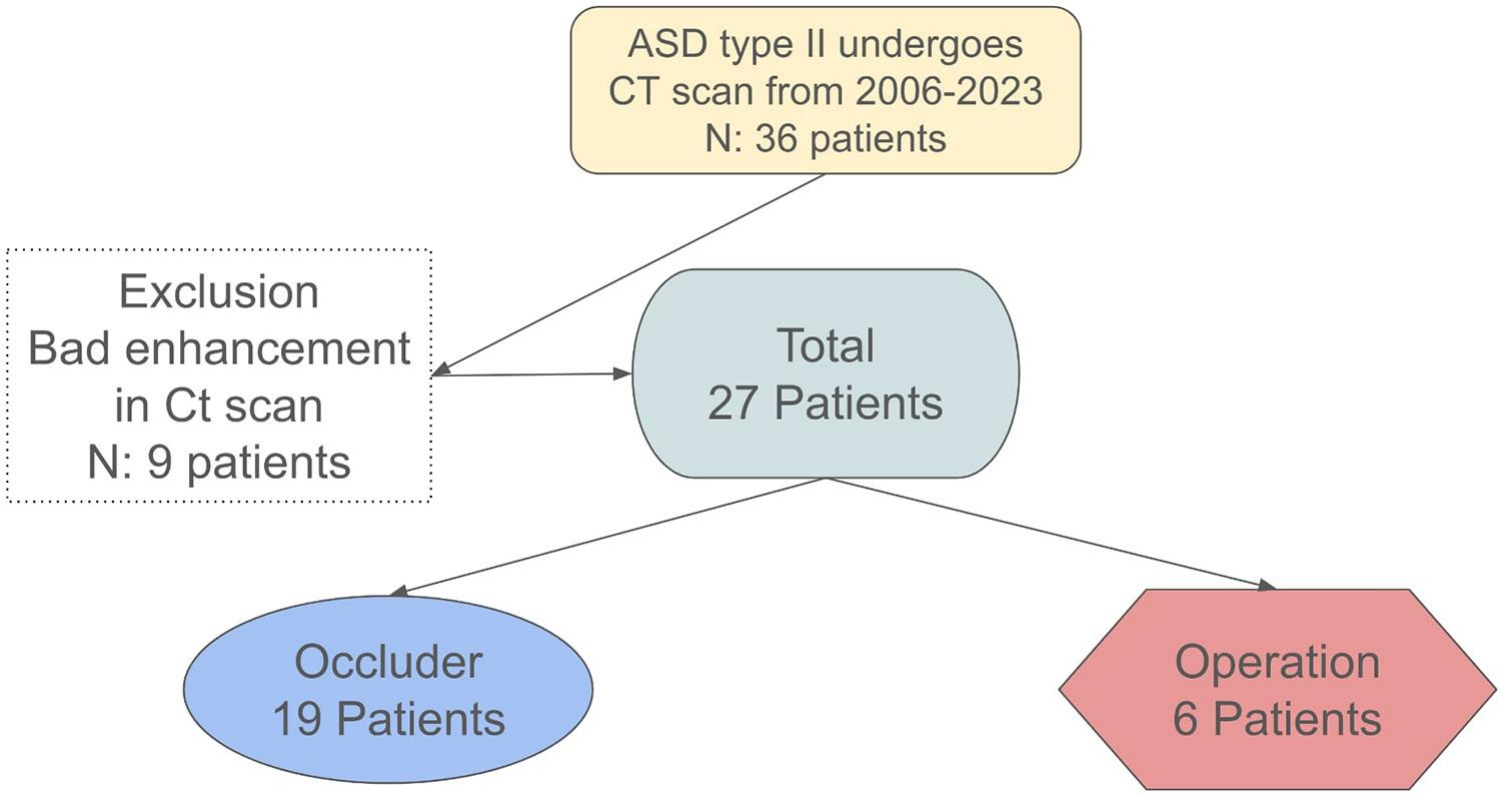
Patient selection flow chart.

Figure 1 illustrates the patient selection process for this retrospective study. A total of 36 patients with secundum-type atrial septal defects (ASD type II) underwent multidetector computed tomography (MDCT) between 2006 and 2023. Nine patients were excluded due to inadequate CT image quality caused by poor contrast enhancement.

The final study cohort comprised 27 patients, of whom 19 underwent successful transcatheter occluder placement and 6 proceeded to surgical repair.

### Study design

This study was a retrospective analysis of pre-existing data, conducted without direct patient interaction or interventional procedures. To ensure compliance with ethical standards, all patient data were fully anonymized, safeguarding confidentiality throughout the research process. The research protocol was approved by the local human research committee. A retrospective review was conducted on patients with type II atrial septal defects (ASDs) who underwent CT scans before receiving transcatheter ASD occluders. We used volume rendering to create a CT virtual cardioscope. Initially, on a Syngo MultiModality Workplace workstation by Siemens, four-chamber and two-chamber MultiPlanar Reconstruction (MPR) views were obtained to determine the plane and center of the ASD. Subsequently, a line perpendicular to the plane and passing through the center of the ASD was identified. Virtual cardioscope images were then generated using the “fly-through” technique, navigating along this line: commencing from the right atrium, passing through the ASD, and concluding in the left atrium.

In virtual cardioscopy, the interatrial septum (IAS) was systematically divided into four quadrants when viewed from the right atrial perspective. This quadrant classification employed a standardized 45 degrees rotational alignment relative to the IAS to ensure consistent and reproducible visualization of the septal rims across patients.

The interatrial septum is a complex three-dimensional, curved structure, and minor variations in viewing angle during virtual cardioscopy or transesophageal echocardiography (TEE) can substantially alter the apparent length and morphology of the surrounding septal rims. Such variability may lead to inconsistent rim assessment, which is critical for determining suitability for transcatheter device closure. To address this challenge, a fixed 45 degrees rotational orientation was applied to normalize the viewing plane, thereby minimizing the effects of patient-to-patient differences in cardiac orientation and atrial geometry.

Using this standardized orientation, an imaginary cross was positioned at the center of the IAS and rotated 45 degrees clockwise. The four quadrants were then defined according to their anatomical relationships: quadrant 1 opened toward the ostium of the superior vena cava (SVC), quadrant 2 toward the tricuspid annulus, quadrant 3 toward the ostium of the inferior vena cava (IVC), and quadrant 4 toward the right lateral dorsal wall of the right atrium. These quadrants were arranged sequentially in a clockwise direction.

This rotation ensures that when clinicians measure the size of the septal rim tissue remaining in each of these four quadrants, their measurements are consistent and reproducible regardless of how the patient's heart is naturally positioned in the chest. This is crucial for determining if the defect has enough surrounding tissue (the “rims”) to safely close it using a catheter-based device.

A circle was fitted to the shape of the ASD, and the region with insufficient rim tissue was identified by measuring the angle to the center of the circle, referred to as the “rimless angle.” We measured the rimless angle for each patient and utilized this angle to indicate rim adequacy. The directions of the extension of these ASDs were also recorded, especially for their rimless parts. Procedural outcomes were categorized as successful or unsuccessful based on the final result of the attempted transcatheter atrial septal defect (ASD) closure. A successful case was defined as one in which the occlusion device was correctly deployed, remained stably positioned after release, and achieved effective defect closure without procedure-related complications. An unsuccessful case was defined as failure of transcatheter closure, including inability to achieve stable device positioning, device embolization, or abandonment of the procedure with subsequent conversion to surgical repair due to inadequate device fit or insufficient septal rim support. All MDCT measurements were performed independently. In one instance, a senior observer appointed by the two participating centers (“observer 1″) with three to twenty years of experience in cardiac imaging interpreted the CT examinations. On two occasions, observer 2, a novice radiologist with five years of experience in cardiac imaging, conducted retrospective observations in a blinded manner.

### Statistical analysis and power evaluation

A retrospective descriptive study was conducted, and statistical analysis was performed. Parametric data are expressed as mean ± SD or number in percentage. An independent T-test was used to compare means. Non-parametric analysis was performed using the Chi-square test. IBM SPSS 23.0 for Windows was used for the analysis. *P*-value of <0,05 was considered statistically significant.

Given the study's limited sample size (24 patients), a *post-hoc* power analysis was conducted to assess the robustness of the statistical results. Specifically, the analysis focused on the comparison of rimless angles between the occluder and surgery groups. The effect size was calculated using the following formula:

Effect Size (Cohen's d).

$$d=\frac{Mean1-Mean\;2}{Pooled\;Standard\;Deviation}$$

Where pooled standard deviation is calculated as:

Pooled Standard Deviation=$\surd \frac{(n1-1)\;.\;SD_{1}^{2}+(n2-1).\;SD_{2}^{2}}{n1+n2-2}$

Substituting the values from our study:

- Mean_1_ = 41.47° (occluder group), Mean_2_ = 109.45° (operation group)
- SD_1_ = 32.36°, SD_2_ = 38.83°
  n1=18,n2=6
  $$\;\begin{array}{c} Pooled\;Standard\;Deviation\;=\;\surd \frac{(18-1)\;.\;(32.36{)}^{2}+(6-1).\;(38.83{)}^{2}}{18+6-2}=33.94^{\circ }\;\\ d=\frac{41.47-109.45}{33.94}=\;-2.00\; \end{array}$$

The effect size (Cohen's d) was calculated to be −2.00, indicating a very large difference between the groups. Using a pooled standard deviation of 33.94 degrees and sample sizes of 18 and 6 patients for the occluder and surgery groups, respectively, the achieved power was determined to be 98.2% at a significance level of 0.05.

This high power demonstrates that the study was adequately powered to detect meaningful differences in rimless angles between the groups, assuring credibility to the observed results despite the small sample size.

## Result

**Figure 2:**
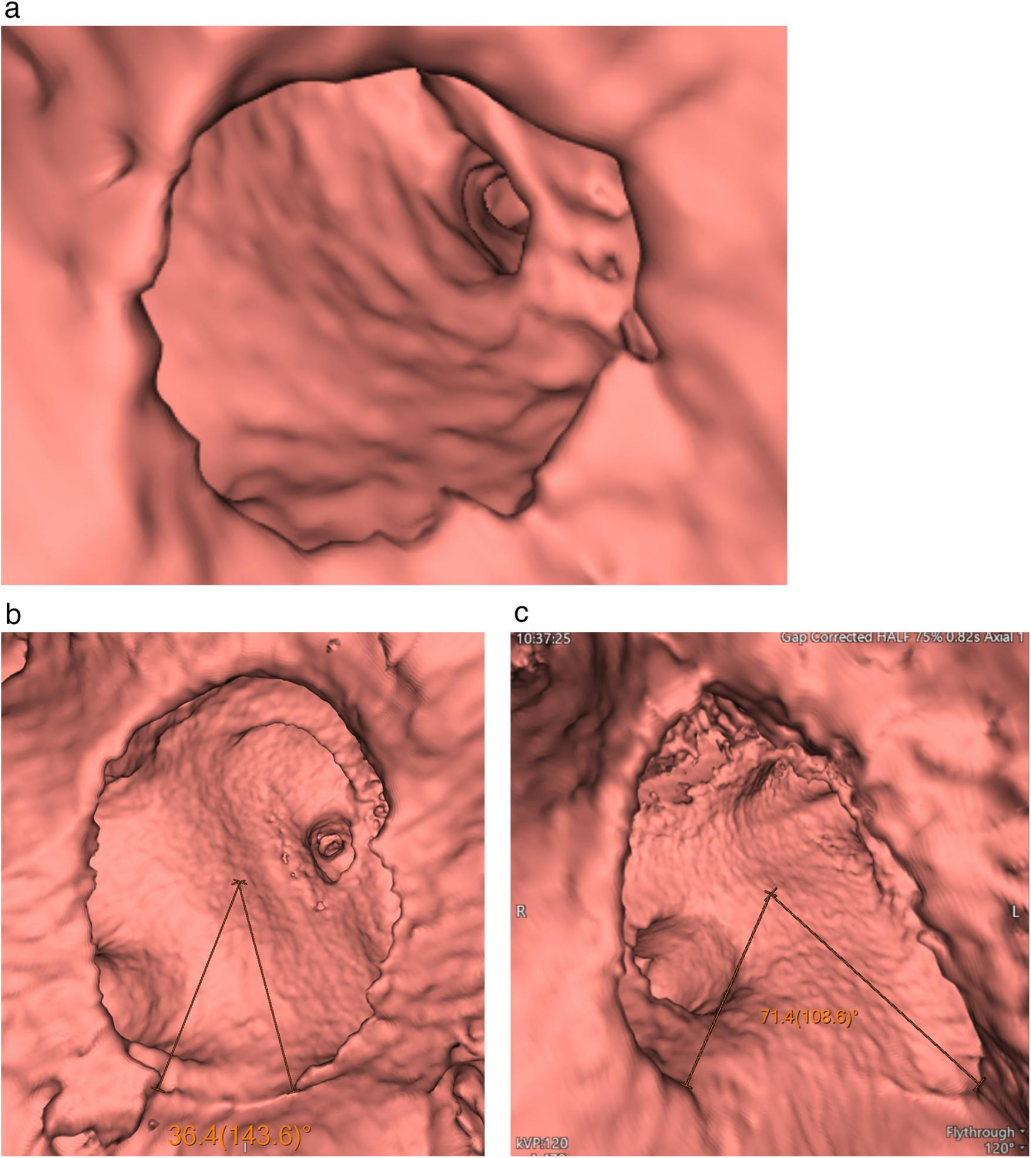
Virtual cardioscope shows a rimless degree **(a)**, a small rimless degree in quadrant 3 **(b)**, and a large rimless degree in quadrant 3 **(c).**

A total of 24 patients with secundum atrial septal defect (ASD) were observed from January 2007 to April 2023 (Table 1) and were evaluated by virtual cardioscope (Figures 2a–c). Eighteen patients underwent successful occlusion therapy for ASD without complications. Nine out of the 18 patients were females and ages ranging from 1 year to 60 years old. Three patients exhibited multiple defects in quadrants I and II, with rimless angles approximately 52.6 degrees in quadrant I and 29 degrees in quadrant II, and in quadrants II and III, with rimless angles around 18.1 degrees in quadrant I and 32.9 degrees in quadrant III. Additionally, one patient had multiple defects in quadrants I to II with a rimless angle of 61.7 degrees and quadrants II to III with a rimless angle of 43.1 degrees. The largest rimless angle among the successfully managed occluder group was approximately 85.2 degrees. The largest defect size along the long axis was about 3.77 cm and along the short axis was 6.77 cm, with a rimless angle of 76.1 degrees in quadrants I and II, successfully managed using an occluder.

**Table 1:** Comparisons of demographic and baseline clinical data between patients in occluder and operation group.

Characteristics	Occluder (*n* = 18)	Operation (*n* = 6)	*P*-value test
Female	9 (50%)	4 (66.7%)	0.478
Age	23.05 ± 22.34 (range 1–60)	15.58 ± 16.26 (range 1–41)	0.461
MDCT Measurement			
Rimless Angle (degree)	41.47 ± 32.36 (range 0–85.2)	109.45 ± 38.83 (range 73.3–175.4)	<0.001*
Long Axis (cm)	1.92 ± 0.68 (range 1.1–3.7)	2.1 ± 0.53 (range 0–85.2)	0.568*
Short Axis (cm)	3.10 ± 1.33 (range 1.6–6.7)	2.9 ± 1.36 (range 1.72–5.3)	0.813*
Location and Extension			
Quadrant I	0 (0%)	0 (0%)	-
Quadrant II	2 (11.1%)	0 (0%)	0.394
Quadrant III	4 (22.2%)	3 (50%)	0.195
Quadrant IV	1 (5.6%)	0 (0%)	0.555
More than 1 Quadrant	5 (27.9%)	3 (50%)	0.317
Multiple Sites	3 (17.1%)	0 (0%)	0.285

Data are presented as mean ± SD or number (percentage).

* *p*-value of Independent *T*-test.

Six patients underwent surgery to close the interatrial defect. Four patients were females and age ranging from 1 year to 41 years old. Three out of these six patients were failed occlusion attempts. These patients had rimless angles of 73.3 degrees in quadrant III with defect sizes of approximately 2.18 cm along the long axis and 5.3 cm along the short axis. The second patient had rimless angles of 73.3 degrees in quadrants III and IV with defect sizes of approximately 1.69 cm along the long axis and 3.51 cm along the short axis. Meanwhile, the third patient had a large rimless angle of approximately 116.6 degrees in quadrant III with a long axis angle of 2.03 cm and a short axis angle of 2.24 cm.

The largest rimless angle in the surgery group was around 175.4 degrees. The largest defect along the long axis was approximately 2.96 cm and along the short axis was 5.3 cm in a different patient. The majority of defects found in the surgery group were located in quadrant III, with three patients having rimless angles ranging from 164.5 degrees to 73.3 degrees, and defects spanning quadrants III and IV in three patients, with rimless angles ranging from 167.6 degrees to 73.3 degrees. This differs slightly from the occluder-managed group, where approximately four patients had defects in quadrant III, with the largest defect angle being approximately 70.4 degrees.

The occluder-managed group had defect locations in quadrants I and II in one patient, quadrant II in two patients, quadrants II and III in three patients, quadrants III and IV in one patient, and quadrant IV in one patient. Based on these findings, it is interesting to note that the surgery-managed group predominantly had defects in quadrants III and IV, with a minimum angle of approximately 73.3 degrees, while the maximum angle in the occluder-managed group was around 50.9 degrees in quadrants III and IV.

The comparisons of demographic and baseline clinical data between the 18 patients who undergone transcathether approach and 6 patients with surgical approach are listed in Table 1. Patients who undergone transcatheter approach were older than patients in the surgical group. There were no differences between the 2 groups in regard to long axis and short axis in MDCT (all *p* > 0.05). However, the rimless angle in the operation group are larger than the operation group and there were differences between the 2 groups (*p* < 0,05). Location and extension of the defect did not have significant association in determining treatment procedure. The findings of this study are highly intriguing, providing valuable insights into how the magnitude of rimless angles and defect locations can influence the success of occluder management.

## Discussion

The dataset comprising 24 patients provide valuable insights into the interventions conducted and their implications for patient care and research. The near-even gender distribution observed in the study, with 11 males and 13 females undergoing interventions, suggests a diverse group of patients participated in the study. This balanced representation of males and females indicates that the interventions were not biased towards a particular gender, enhancing the generalizability of the findings to a broader patient population. Including a diverse group of patients in research studies is essential for ensuring that findings can be applied more broadly and effectively in clinical practice, as it allows for a more representative sample that better reflects the diversity of patients typically encountered in medical practice.

Moreover, the diverse age range of patients at the time of intervention, spanning from 1 year to 60 years old, highlights the inclusivity of the study population across various age groups. The dataset's broad spectrum of ages suggests that the interventions were not restricted to a specific age demographic but encompassed a wide range of patients with potentially unique medical considerations and treatment requirements. Understanding how interventions impact patients across different age groups is crucial for tailoring treatment strategies to individual needs and optimizing patient outcomes. Additionally, considering the potential differences in disease presentation and treatment response across age groups is vital for providing personalized and effective care to patients of all ages.

The treatment landscape for atrial septal defects (ASDs) has undergone significant evolution over recent decades, with a notable shift towards percutaneous transcatheter closure as the preferred approach for managing secundum-type ASDs. This transformation has been driven by advancements in medical technology and the development of innovative devices and techniques, leading to improved efficacy and safety outcomes compared to traditional surgical closure methods. Our study contributes to this evolving field by providing insights into the outcomes and challenges associated with both percutaneous and surgical interventions for secundum ASDs.

Our findings highlight several key observations regarding the management of secundum ASDs. Firstly, we observed that percutaneous closure demonstrated favorable outcomes, with a majority of patients achieving successful closure without complications. This aligns with previous studies indicating the effectiveness of percutaneous transcatheter closure in treating secundum ASDs and its superiority over surgical repair in terms of early complications and long-term outcomes.

### Limitations and strengths

Despite the overall success of percutaneous closure, our study also identified challenges and potential complications associated with this approach. Specifically, we encountered cases involving complex lesions with multiple defects, rim deficiencies, or large defect sizes, which posed unique obstacles for interventionists. These findings accentuate the importance of careful patient selection and expertise in navigating complex anatomical variations to ensure optimal outcomes for patients undergoing percutaneous closure.

Furthermore, our study underline the critical role of accurate morphological assessment in guiding treatment decisions for ASDs. Factors such as defect size, location, and rim adequacy play a crucial role in determining the suitability of either percutaneous or surgical intervention. Our use of multidetector computed tomography (MDCT) and virtual cardioscopy allowed for detailed anatomical evaluation, enabling us to assess defect characteristics and identify potential obstacles to successful closure. This comprehensive approach to pre-procedural planning is essential for optimizing patient outcomes and minimizing the risk of complications.

Our study face the challenges posed by small sample sizes, retrospective design and the inherent limitations of current imaging modalities, particularly transesophageal echocardiography (TEE), in assessing atrial septal defects (ASDs). While TEE remains an essential tool for guiding interventional procedures, its constraints become apparent in cases involving large defects or complex morphologies, where detailed anatomical visualization is crucial. These limitations underline the potential value of alternative imaging techniques, such as multidetector computed tomography (MDCT), which can provide more comprehensive anatomical information and enhance procedural planning. Despite the small sample size, the study's high statistical power punctuate the credibility of its findings, particularly the observed differences in rimless angles between groups. This suggests that the study was adequately powered to detect meaningful variations, lending robustness to the conclusions. Nevertheless, larger-scale studies are needed to validate these results and further investigate additional parameters.

### Future directions

Prospective, larger-scale studies are warranted to validate these findings and further refine imaging criteria for transcathether vs. surgical selection. In particular, prospective multi-modality comparisons involving MDCT, 2D TTE, 2D TEE, and 3D TEE would provide stronger validation of virtual cardioscopy and clarify its complementary role in routine ASD assessment. Future work should also explore additional quantitative parameters and the development of standardized virtual views to improve reproducibility. Ultimately, integrating virtual cardioscopy into a comprehensive pre-procedural imaging workflow may optimize patient selection and procedural outcomes.

## Conclusion

In conclusion, our study contributes to the growing body of evidence supporting the efficacy and safety of percutaneous transcatheter closure for secundum ASDs. By highlighting the importance of accurate morphological assessment and the challenges associated with complex lesions, our findings underscore the need for continued innovation and refinement in ASD treatment strategies. Ultimately, our goal is to provide the best possible care for patients with ASDs, ensuring optimal outcomes and quality of life.

## Data Availability

The raw data supporting the conclusions of this article will be made available by the authors, without undue reservation.
